# Finite difference magnetoelastic simulator

**DOI:** 10.12688/openreseurope.13302.1

**Published:** 2021-04-19

**Authors:** Frederic Vanderveken, Jeroen Mulkers, Jonathan Leliaert, Bartel Van Waeyenberge, Bart Sorée, Odysseas Zografos, Florin Ciubotaru, Christoph Adelmann

**Affiliations:** 1 Imec, Leuven, 3001, Belgium; 2 Departement Materiaalkunde, SIEM, KU Leuven, Leuven, 3001, Belgium; 3 Departement Vastestofwetenschappen, DyNaMat, Universiteit Gent, Gent, 9000, Belgium; 4 Departement Elektrotechniek, TELEMIC, KU Leuven, Leuven, 3001, Belgium; 5 Departement Fysica, Universiteit Antwerpen, Antwerpen, 2000, Belgium

**Keywords:** magnetoelasticity, magnetoelectricity, micromagnetics, finite differences

## Abstract

We describe an extension of the micromagnetic finite difference simulation software MuMax3 to solve elasto-magneto-dynamical problems. The new module allows for numerical simulations of magnetization and displacement dynamics in magnetostrictive materials and structures, including both direct and inverse magnetostriction. The theoretical background is introduced, and the implementation of the extension is discussed. The magnetoelastic extension of MuMax3 is freely available under the GNU General Public License v3.

## Plain language summary

Magnets are common items in daily life. Their applications range from fridge magnets to remember recent holidays to large magnets in the motors of electric cars. Magnets are typically made of ferromagnetic materials, such as iron or cobalt, that possess a spontaneous magnetization. Magnets generate magnetic fields in their surroundings, which lead to magnetic forces that can be felt when two magnets attract each other. A special property of magnets that is less known is that the magnetization is also coupled to their shape. This effect is called magnetostriction. Hence, when one changes the magnetization direction, this also leads to a shape change of the magnet, for example to the contraction of a bar magnet. Also, an inverse effect exists that can change the magnetization direction when a magnet is deformed. Together, these effects can be classified as magnetoelastic. Magnetostriction is typically rather small, with relative length changes between 1 and 1000 parts per million, depending on the material. Despite the small effect, it has been employed for example for ultrasound generation and detection in Sonar applications that are used for navigation of ships and submarines.

Recently, researchers have also tried to apply magnetostriction in microelectronics. Magnetic hard disk drives have been used in computers for decades but the attempt to make computer chips with magnetic materials is a much more recent development. In some advanced technologies that may replace today’s CMOS transistors in the future, magnetoelastic effects play key roles. To better understand the operation of such chips, it is important to be able to simulate their magnetoelastic behavior. However, magnetoelastic simulation software packages are still scarce, especially as open source. Here, we describe such a software, which is available under the GNU General Public License, and give a short introduction on how to use it.

## Introduction

Spintronic applications have attracted ever increasing interest in the last decades, following the commercial success of hard disk drives. More recently, magnetic random access memory (MRAM) has been developed ^1– 3^ and integrated as embedded memory in commercial microelectronic systems ^4– 6^. Beyond these applications, many potential alternative spintronic device concepts have been researched ^6– 12^, including magnetic sensors ^13, 14^ and spintronic logic ^15– 19^.

**Figure 1.**
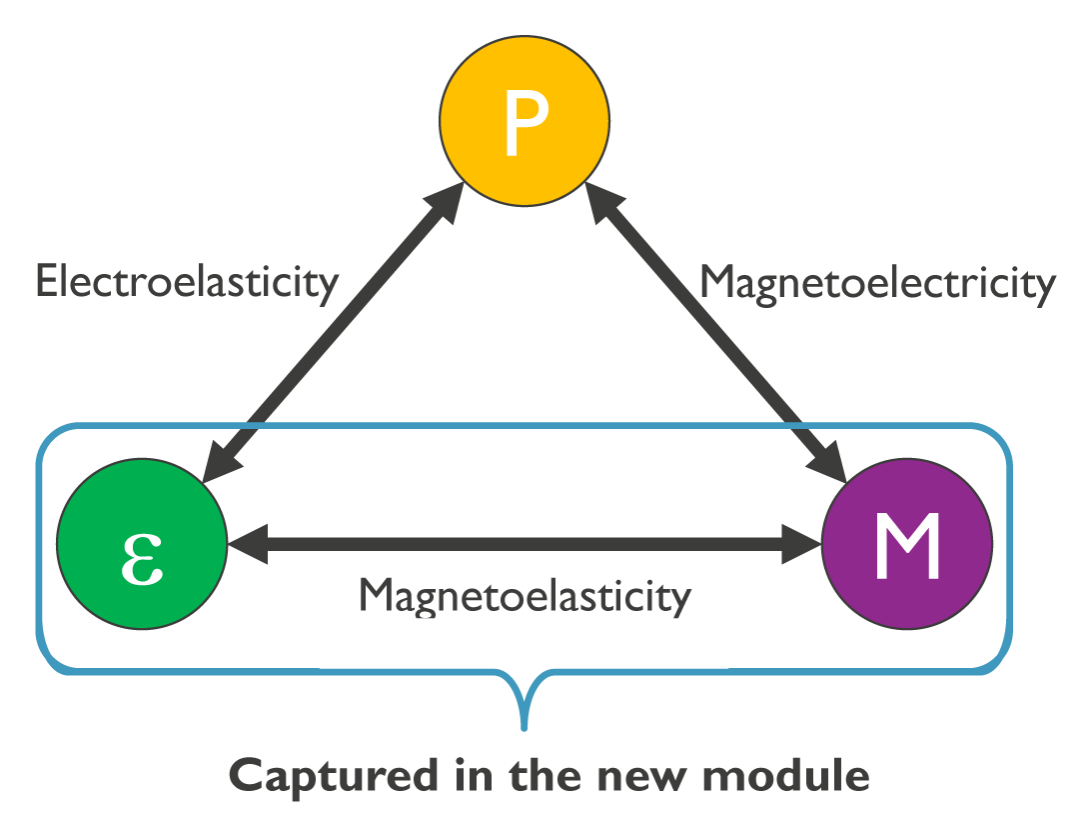
Schematic of the magnetoelectric coupling in magnetoelectric composite materials, in which the coupling between dielectric polarization *P* and magnetization *M* occurs indirectly via strain *ε*. The software described here allows for the numerical simulation of the magnetoelastic coupling in complex structures.

Magnetoelectrics are a class of spintronic materials that have received particular attention due to their potential to manipulate magnets at very low power and the possibility for new functionalities ^20– 24^. Besides multiferroic materials ^25– 29^, which possess simultaneous magnetic and ferroelectric polarization, magnetoelectric composites have been at the center of research on magnetoelectricity for decades ^20, 23, 29– 34^. Such composites consist of piezoelectric and magnetostrictive materials and rely on the interaction between the magnetization and mechanical degrees of freedom. Applying an electric field to a piezoelectric material leads to the generation of a mechanical deformation (strain) that can be transferred to an adjacent magnetostrictive material. The strain inside the (ferromagnetic) magnetostrictive material leads then to a magnetoelastic effective magnetic field via the Villari effect (inverse magnetostriction) that can exert a torque on the magnetization. Conversely, the rotation of the magnetization in a magnetostrictive material generates strain that can lead to charge separation and an electric polarization in an adjacent piezoelectric material. This indirect coupling between electric fields and magnetization is schematically represented in Figure 1. This paper (and the software described therein) addresses the magnetoelastic interaction between strain *ε* and magnetization *M*.

For many applications, in particular in microelectronics, spintronic devices must be miniaturized to the nanoscale to be competitive. The integration of magneto-electric composites into scaled spintronic devices requires the understanding of the piezoelectric as well as the magnetoelastic interactions at the nanoscale. While the fundamental magnetoelectric and magnetoelastic coupling is well understood ^35– 40^, the detailed behavior of nanoscale structures has only very recently been addressed ^41– 48^. Nanoscale spintronic devices may possess complex structures with different functional areas and materials, leading to spatially varying properties and highly convoluted boundary conditions. It is clear that such devices cannot be represented well by (often approximate) analytical models and that numerical simulations are typically necessary.

While many commercial and free solutions exist to simulate nanoscale piezoelectric devices due to the importance of microelectromechanical systems (MEMS), no comprehensive free software package exists to date to simulate magnetoelastic interactions at the nanoscale. At microwave frequencies in the GHz range, which are often relevant for spintronic applications, the magnetization dynamics become nontrivial and micromagnetic simulators are required to describe and understand the device performance. While it is possible to include magnetoelastic effective fields in micromagnetic simulations using the open-source simulator OOMMF ^49– 51^, M uM ax3 ^52^, or Boris ^53^, such approaches cannot describe the backaction of the magnetodynamics on the elastodynamics of the system. Hence, a full description of the magnetoelastic dynamics of such a system requires solve simultaneously both the elastodynamic and magnetodynamic equations of motion. Whereas full magnetoelastic simulations of nanoscale structures have been published using C omsol ^®^, ^54– 62^ the software modules developed for these simulations are not freely available for the scientific community.

In this paper, we present a magnetoelastic software module that allows for the simultaneous numerical solution of magneto- and elastodynamics in complex geometries, including both direct and inverse magnetostrictive interactions. The module is written as an extension of the widely used micromagnetic solver M uM ax3 ^52^. Both the solver and the extension are available under the GNU General Public License v3. This approach allows for the usage of all pre-defined functionalities of M uM ax3 in combination with the magnetoelastic module. Furthermore, simulations are accelerated using GPUs with respect to standard simulators that run on CPUs.

## Theoretical background

The magnetoelastic software module described below extends the well-known micromagnetic solver M uM ax3 and allows for the simulation of the magnetoelastodynamic behavior in magnetostrictive materials. In this section, the fundamental underlying equations are introduced, which are implemented in the solver.

The magnetization dynamics are described by


$$\;\dot{m}=T\;(1)$$

with ****m**** = ***M*** / *M _s_* the normalized magnetization, *M _s_* the saturation magnetization, and ****T**** the magnetic torque. The dot denotes a time derivative. In the absence of spin currents, the torque is equal to the Landau-Lifshitz torque, given by


$$\;T=\frac{-\gamma_{0}}{1+\alpha^{2}}(m\;\times H_{\text{eff}}+\alpha (m\;\times (m\;\times H_{\text{eff}})))\;,\;(2)$$

with γ _0_ = *µ*_0_γ, γ the absolute value of the gyromagnetic ratio, *α* the phenomenological damping constant, and ****H****_eff_ the effective magnetic field. This effective field contains all effects that influence the magnetization dynamics, including exchange and dipolar interactions. Equation (1) and Equation (2) form the base of the micromagnetic solver already implemented in M uM ax3.

Effects of inverse magnetostriction can be inserted into the above framework by adding a magnetoelastic field ****H****_mel_ to the effective field ****H****_eff_ in Equation (2). For materials with cubic (or higher) crystal symmetry, the magnetoelastic effective field is given by 63– 65


$$\;H_{\text{mel}}=-\frac{2}{\mu_{0}M_{s}}\left[\begin{array}{c} B_{1}\varepsilon_{\text{XX}}m_{x}+B_{2}(\varepsilon_{\text{XY}}m_{y}+\varepsilon_{zx}m_{z})\\ B_{1}\varepsilon_{\text{YY}}m_{y}+B_{2}(\varepsilon_{\text{XY}}m_{x}+\varepsilon_{yz}m_{z})\\ B_{1}\varepsilon_{\text{ZZ}}m_{z}+B_{2}(\varepsilon_{\text{ZX}}m_{x}+\varepsilon_{yz}m_{y}) \end{array}\right]\;,\;(3)$$

with *B*_1_ and *B*_2_ the first and second magnetoelastic coupling constants, respectively, and *ε _ij_* the components of the mechanical strain tensor $\hat{\varepsilon }$ .

The elastodynamics in a solid are described by the second order differential equation ^66, 67^

$$\;\rho \ddot{u}+\eta \dot{u}=f_{\text{eff}},\;(4)$$

or equivalently by two first order differential equations

$$\;\begin{array}{l} \dot{u}=v\\ \dot{v}=a=\frac{f_{\text{eff}}-\eta v}{\rho }, \end{array}\;(5)$$

with ***u*** the displacement, ***v*** the velocity, ***a*** the acceleration, *ρ* the mass density, *η* a phenomenological damping parameter, and ***f***_eff_ the effective body force. This body force contains all effects that influence the displacement dynamics, such as elastic or magnetoelastic contributions. These two contributions are the ones considered in this extension. The elastic body force is given by ***f***_el_ = ∇ $\hat{\sigma }$ , with $\hat{\sigma }$ the mechanical stress tensor. For small displacement values, Hooke’s law $\hat{\sigma }=\hat{c}\hat{\varepsilon }$ is valid. Here, $\hat{c}$ is the fourth order stiffness tensor. Together with the definition of the strain $\hat{\varepsilon }=\frac{1}{2}\left(\nabla u+{\left(\nabla u\right)}^{T}\right)$ , the elastic body force for materials with cubic crystal structure (or higher symmetry) can be written as

$$f_{\text{el}}=\left[\begin{array}{l} \frac{\partial }{\partial x}\left[c_{11}\frac{\partial u_{x}}{\partial x}\right]+\left(\frac{\partial }{\partial y}\left[c_{44}\frac{\partial u_{x}}{\partial y}\right]+\frac{\partial }{\partial z}\left[c_{44}\frac{\partial u_{x}}{\partial z}\right]\right)+\left(\frac{\partial }{\partial y}\left[(c_{12}+c_{44})\frac{\partial u_{y}}{\partial x}\right]+\frac{\partial }{\partial z}\left[(c_{12}+c_{44})\frac{\partial u_{z}}{\partial x}\right]\right)\\ \frac{\partial }{\partial y}\left[c_{11}\frac{\partial u_{y}}{\partial y}\right]+\left(\frac{\partial }{\partial x}\left[c_{44}\frac{\partial u_{y}}{\partial x}\right]+\frac{\partial }{\partial z}\left[c_{44}\frac{\partial u_{y}}{\partial z}\right]\right)+\left(\frac{\partial }{\partial x}\left[(c_{12}+c_{44})\frac{\partial u_{y}}{\partial y}\right]+\frac{\partial }{\partial z}\left[(c_{12}+c_{44})\frac{\partial u_{z}}{\partial y}\right]\right)\\ \frac{\partial }{\partial z}\left[c_{11}\frac{\partial u_{z}}{\partial z}\right]+\left(\frac{\partial }{\partial x}\left[c_{44}\frac{\partial u_{z}}{\partial x}\right]+\frac{\partial }{\partial y}\left[c_{44}\frac{\partial u_{z}}{\partial y}\right]\right)+\left(\frac{\partial }{\partial x}\left[(c_{12}+c_{44})\frac{\partial u_{x}}{\partial z}\right]+\frac{\partial }{\partial y}\left[(c_{12}+c_{44})\frac{\partial u_{y}}{\partial z}\right]\right) \end{array}\right]\;.\;(6)$$

Here, *c*_ij_ are the components of the stiffness tensor in reduced dimensionality, *i.e.* in Voigt notation.

Considering uniform displacement along the *z*-direction, *i.e.* *∂ **u***/ *∂z* = 0, Equation (6) can be simplified to

$$\;f_{\text{el}}=\left[\begin{array}{c} \frac{\partial }{\partial x}\left[c_{11}\frac{\partial u_{\text{x}}}{\partial x}\right]+\frac{\partial }{\partial y}\left[c_{44}\frac{\partial u_{\text{x}}}{\partial y}\right]+\frac{\partial }{\partial y}\left[(c_{12}+c_{44})\frac{\partial u_{\text{y}}}{\partial x}\right]\\ \frac{\partial }{\partial y}\left[c_{11}\frac{\partial u_{\text{y}}}{\partial y}\right]+\frac{\partial }{\partial x}\left[c_{44}\frac{\partial u_{\text{y}}}{\partial x}\right]+\frac{\partial }{\partial x}\left[(c_{12}+c_{44})\frac{\partial u_{\text{x}}}{\partial y}\right]\\ \frac{\partial }{\partial x}\left[c_{44}\frac{\partial u_{\text{z}}}{\partial x}\right]+\frac{\partial }{\partial y}\left[c_{44}\frac{\partial u_{\text{z}}}{\partial y}\right] \end{array}\right]\;.\;(7)$$

This form allows for the description of the elastodynamics in 1D, 2D, and quasi-3D elastic systems.

In magnetostrictive materials, there is also a body force contribution due to the magnetostriction effect. For a magnetostrictive material with cubic crystal structure (or higher symmetry), the magnetoelastic body force is given by

$$f_{\text{mel}}=2B_{1}\left[\begin{array}{l} m_{x}\frac{\partial m_{x}}{\partial x}\\ m_{y}\frac{\partial m_{y}}{\partial y}\\ m_{z}\frac{\partial m_{z}}{\partial z} \end{array}\right]+B_{2}\;\left[\begin{array}{l} m_{x}\left(\frac{\partial m_{y}}{\partial y}+\frac{\partial m_{z}}{\partial z}\right)+m_{y}\frac{\partial m_{x}}{\partial y}+m_{z}\frac{\partial m_{x}}{\partial z}\\ m_{y}\left(\frac{\partial m_{x}}{\partial x}+\frac{\partial m_{z}}{\partial z}\right)+m_{x}\frac{\partial m_{y}}{\partial x}+m_{z}\frac{\partial m_{y}}{\partial z}\\ m_{z}\left(\frac{\partial m_{x}}{\partial x}+\frac{\partial m_{y}}{\partial y}\right)+m_{x}\frac{\partial m_{z}}{\partial x}+m_{y}\frac{\partial m_{z}}{\partial y} \end{array}\right]\;.\;(8)$$

The mechanical boundary conditions at the surface are

$$\;F_{s}=\hat{\sigma }\;n,\;(9)$$

with ***F***_s_ the traction force per unit surface and ***n*** the surface normal.

## Methods

In this section, we describe the M uM ax3 framework as well as the implementation of the magnetoelastic module. The integration of the module into M uM ax3 has the benefit that all predefined M uM ax3 functions can also be applied to the magnetoelastic extension. The first part of this section explains the numerical implementation of the elastodynamic equations as well as the elastic body force. This is followed by an overview of all novel functions and parameters defined in the magnetoelastic extension together with instructions on how to use them.

### M uM ax3 framework

M uM ax3 is an established finite difference micromagnetic solver. In the following, several important aspects of the M uM ax3 framework as well as the procedures for running simulations and extracting data are briefly introduced. This introduction is meant to provide the necessary basis to properly use the magnetoelastic module in a later stage.

In M uM ax3, simulations are often defined by a script with the .mx3 extension that contains all information necessary to describe the system under study. The script syntax is based on a subset of the Go language and is comprehensively explained in the M uM ax3 documentation ^52, 68^. The most important aspects are, however, briefly explained here.

The first part of the script contains typically information about the dimensions of the simulated system and the mesh size. The user sets the mesh and the dimensions by defining the cell size together with the number of cells in the three orthogonal directions. The cell shape is always a cuboid. Within the simulated system, it is possible to define different regions with different geometries, in which the material parameters can vary, and which can therefore represent different materials.

Next, material parameters need to be assigned to every region. Based on the provided parameters, M uM ax3 automatically determines which interactions need to be considered in the simulation. Therefore, it is straightforward to include a wide range of different interactions by just specifying appropriate material parameters. Once the mesh with its regions and all material parameters are provided, the simulation can be run in two ways: the first option consists of a numerical energy minimization to determine the equilibrium ground state of the system. The second option consists of the time-dependent integration of the magnetization dynamics using Equation (1). This option allows for the assessment of the temporal evolution of the dynamic variables, in particular the magnetization dynamics. In this paper, we will focus on the second option. Several numerical integration schemes are readily implemented in M uM ax3 and can be selected by the SetSolver(x) command, with x an integer between 1 and 6 ^68, 69^. Once the desired solver is chosen, the simulation can be started by the command run(t_tot), with t_tot the total simulation time set by the user.

M uM ax3 can also perform several mathematical operations on physical variables. For example, this can be useful when extracting and postprocessing the output data. Note that all mathematical operations are performed on GPUs, which typically results in short computational times. All variables can be extracted at any time during the simulation and can be converted to different output formats, such as .ovf, .vtk, or .csv.

### Implementation of the magnetoelastic extension

To simulate the magnetoelastic dynamics, both the magnetic and elastic equations of motion, *i.e.* Equation (1) and Equation (4), respectively, must be solved simultaneously. For the implementation, the two first order differential equations in Equation (5) were chosen instead of the the single second order differential equation in Equation (4) for stability reasons. Hence, the system of equations that must be solved becomes

$$\;\dot{m}=T\;(10)$$

$$\;\dot{u}=v\;(11)$$

$$\;\dot{v}=a=\frac{1}{\rho }\;(f_{\text{eff}}-\eta v),\;(12)$$

which can be written in a compact manner as

$$\;\dot{w}\text{(}t\text{)}=R(t,w),\;(13)$$

with ***w*** = [ *m _x_, m _y_, m _z_, u _x_, u _y_, u _z_, v _x_, v _y_, v _z_*] and ***R*** = [ *T _x_, T _y_, T _z _, v _x_, v _y_, v _z_, a _x_, a _y_, a _z_*]. Note that the magnetic torque ***T*** and the effective body force ****f****_eff_ include the magnetoelastic field and the magnetoelastic body force, respectively, to account for the mutual interactions between the elastic and magnetic domains. Hence, instead of solving a single differential equation, *i.e.* Equation (1), the magnetoelastic module must solve a set of differential equations, including elastic and magnetoelastic interaction terms, as a function of time.

***Time integration*.** The time integration of Equation (13) has been numerically implemented via the fourth order Runge-Kutta (RK4) method given by

$$\;w_{i+1}=w_{i}+\frac{\text{Δ}t}{6}\;(k_{1}+2k_{2}+2k_{3}+k_{4}),\;(14)$$

where the subscript *i* corresponds to the number of the time step with length ∆ *t*. The ****k****-parameters are given by

$$\;\begin{array}{l} k_{1}=R\text{(}t_{i}\text{,}w_{i}\text{)}\;\text{(15)}\\ k_{2}=R\left(t_{i}\;\text{+}\;\frac{\text{Δ}t}{2},w_{i}\;+\frac{\text{Δ}t}{2}k_{1}\right)\;(16)\\ k_{3}=R\left(t_{i}\;\text{+}\;\frac{\text{Δ}t}{2},w_{i}\;+\frac{\text{Δ}t}{2}k_{2}\right)\;(17)\\ k_{4}=R\left(t_{i}\;\text{+}\;\text{Δ}t,w_{i}\;+\text{Δ}tk_{3}\right)\;.\;(18) \end{array}$$

To use this new solver instead of the regular M uM ax3 solvers that integrate only Equation (1), the user must specify SetSolver(9) in the .mx3 script. The regular M uM ax3 solvers run by default with adaptive time stepping. By contrast, the novel solver only runs with a predefined fixed time step ∆ *t*, which also needs to be defined by the user in the .mx3 script. Typical time steps that give accurate results and lead to reasonable simulation times are of the order of ∆ *t ≈* 0.1 ps. The user can also always verify whether the chosen time step is accurate enough by running an additional simulation with a much smaller time step. If both simulations lead to identical results, the chosen time steps are sufficiently small. If the two results differ, the time step should be shortened further.

***Calculation of **R***.** For every time step *i*, the right hand side of Equation (13), ***R*** ( *t, **w***), needs to be calculated several times. The vector ***R*** comprises the magnetic torque ***T***, the velocity ***v***, and the acceleration ***a***. The numerical calculation of ***T*** is already implemented and optimized in the regular M uM ax3 framework. Hence, the calculation of ***T*** is kept untouched in the magnetoelastic extension. The velocity vector ***v*** is obtained by the Runge-Kutta integration of Equation (12). Finally, the acceleration ***a*** is determined by calculating ***a*** = ( ***f***_eff_ *− η* ***v***) */ρ* using the previously found velocity ***v*** in combination with Equation (7) and Equation (8), whose sum is the effective body force.

In the following, the numerical implementation of the elastic body force will be explained in more detail since it is nontrivial in presence of free elastic boundary conditions. The elastic body force depends on second order spatial derivatives of the displacement, as indicated by Equation (6). These spatial derivatives are approximated using the finite difference method. In the bulk of the system, the derivatives are calculated based on the sum of a second order forward and second order backward finite difference using averaged stiffness constants. This is done to properly account for parameters that may vary between different positions (regions). To illustrate this approach, the implemented equations to calculate the second order derivative of ***u*** ( *i, j*) at position ( *i, j*) with respect to *x* and the mixed derivative with respect to *x* and *y* are

$$\frac{\partial }{\partial x}\left[c(i,j)\frac{\partial u(i\;,j)}{\partial x}\right]\approx \frac{c(i\;+1,j)+c(i\;,j)}{2}\frac{u(i\;+1,j)-u(i\;,j)}{\text{Δ}x^{2}}+\frac{c(i\;-1,j)+c(i\;,j)}{2}\frac{u(i\;-1,j)-u(i\;,j)}{\text{Δ}x^{2}};\;(19)$$

$$\begin{array}{l} \frac{\partial }{\partial y}\left[c(i,j)\frac{\partial u(i\;,j)}{\partial x}\right]\approx \frac{c(i\;+1,j\;+1)+c(i-1\;,j\;-1)+c(i+1\;,j\;-1)+c(i-1\;,j\;+1)+4c(i\;,j\;)}{8}\\ \;\times \frac{u(i\;+1,\;j\;+1)\;+\;u(i\;-\;1,\;j\;-\;1)\;+\;u(i\;+\;1,\;j\;-\;1)\;+\;u(i\;-1,\;j\;+\;1)\;}{4\text{Δ}x\text{Δ}y}\;.\;(20) \end{array}$$

The second derivative in the *y*-direction as well as the mixed derivative with respect to *y* and *x* directions are defined in an straightforward analogous way. For uniform stiffness constants, *i.e. c*( *i, j*) *≡ c*, these equations reduce to the regular central finite difference scheme, *i.e.*

$$\frac{\partial }{\partial x}\left[c\frac{\partial u(i\;,j)}{\partial x}\right]\approx c\frac{u(i\;+1,j\;)-2u(i\;,j\;)+u(i\;-1,j)}{\text{Δ}x^{2}}\;(21)$$

and

$$\frac{\partial }{\partial y}\left[c\frac{\partial u(i\;,j)}{\partial x}\right]\approx c\frac{u(i\;+1,j+1\;)+u(i\;-1\;,j-1\;)+u(i\;+1,j-1)+u(i\;-1,j+1)}{4\text{Δ}x\text{Δ}y}.\;(22)$$

***Boundary conditions*.** At the edges of a structure, elastic boundary conditions need to be considered, which alter the differential equations and thus their numerical implementation. Three different elastic boundary conditions are implemented in the magnetoelastic module:

1. Periodic boundary conditions, which connect the left edge to the right edge and the bottom to the top of the structure. These boundary conditions do not represent physical boundaries, and therefore Equation (19) and Equation (20) can be used at the edges as well.
2. Fixed boundary conditions, which also do not require modifications of the bulk finite difference scheme. They can be implemented by defining a region at the boundary with a fixed displacement value ( *e.g.* of 0).
3. Free boundary conditions, which correspond to zero traction force at the surfaces.

The third type of free boundary conditions requires the reformulation of the finite difference scheme at the boundaries to satisfy Equation (9). For simplicity, the simulated structure is assumed to be uniform in the *z*- direction with boundaries only in the *x*- and *y*-directions. Moreover, we consider zero traction force. Then, the Neumann boundary conditions at the free surface are

$$\;\begin{array}{l} \sigma_{xx}n_{x}+\sigma_{xy}n_{y}=0\\ \sigma_{yx}n_{x}+\sigma_{yy}n_{y}=0\\ \sigma_{zx}n_{x}+\sigma_{zy}n_{y}=0. \end{array}\;(23)$$

The free boundary condition in the *x*-direction, *i.e. n _x_* = 1 *, n _y_* = *n _z_* = 0, then becomes *σ _xx_* = *σ _yx_* = *σ _zx_* = 0 and similarly for the *y*-direction, *σ _yy_* = *σ _xy_* = *σ _zy_* = 0.

These expressions must be inserted into the elastic body force at the edges. The implementation of the equations at the mesh edge perpendicular to the *x*-direction is described below as an example. The same procedure can be used to derive expressions for the corresponding equations at the other interfaces.

**Figure 2.**
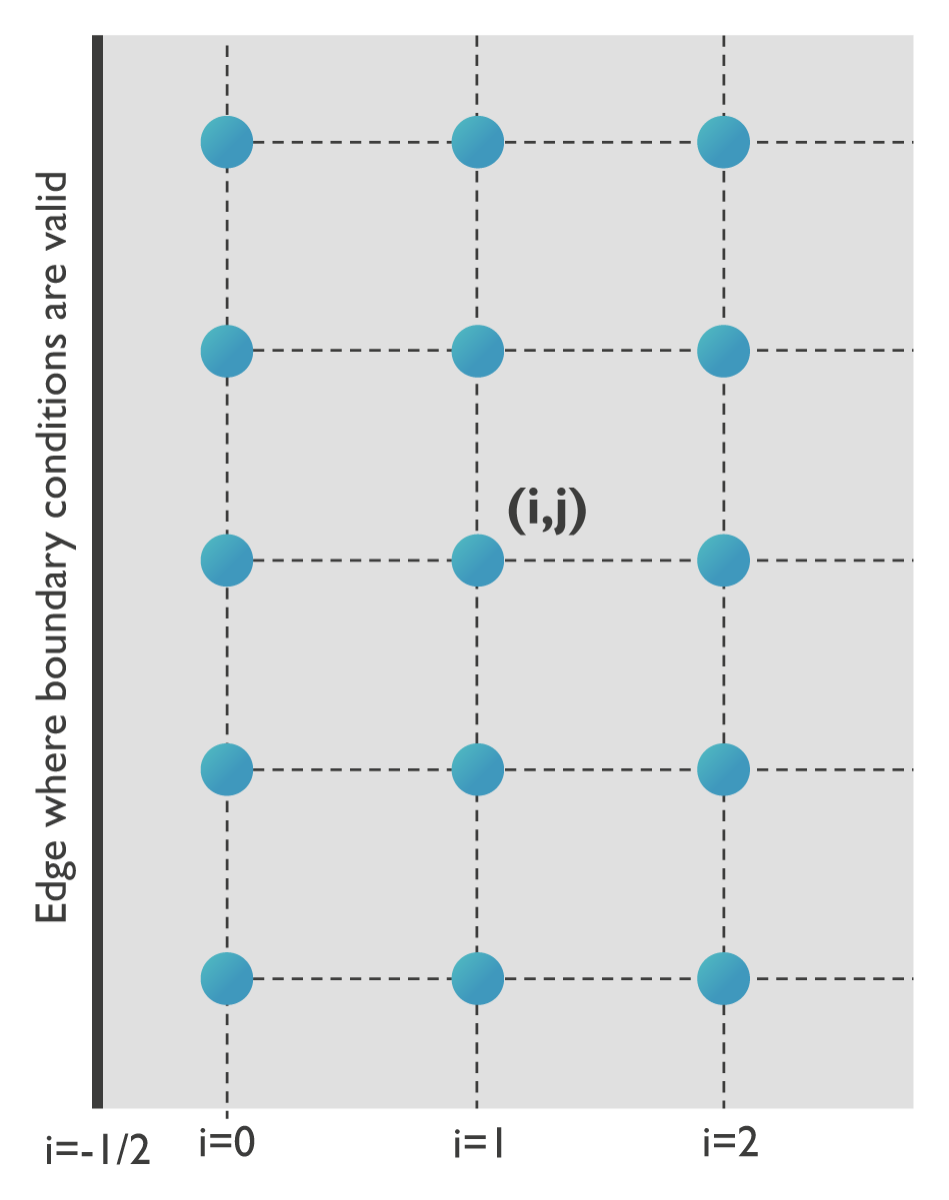
Definition of the grid at a boundary.

The mesh near a boundary in the *x*-direction is visualized in Figure 2. The interface itself is located at positions ( *−*1 */*2 *, j*), which are the exact locations where the boundary conditions in Equation (23) are valid. Note that this boundary also describes the limits of the magnetization, *i.e.* the magnetic material ends at ( *−*1 */*2 *, j*). By contrast, the coordinates of the points next to the boundary edge can be written as (0 *, j*).

For free boundary conditions, the differential equations need to be modified. In the following, the three components of the elastic body force are rewritten in their finite difference approximations taking into account *σ _xx_* ( *i −* 1 */*2 *, j*) = *σ _yx_* ( *i −* 1 */*2 *, j*) = *σ _zx_* ( *i −* 1 */*2 *, j*) = 0.

The *x*-component of the elastic body force is

$$\;f_{\text{el},x}=\frac{\partial \sigma_{xx}(0,j)}{\partial x}+\frac{\partial \sigma_{xy}(0,j)}{\partial y}.\;(24)$$

At location *i* = 0, the first term of Equation (24) is given by

$$\begin{array}{l} \frac{\partial \sigma_{xx}(i=0,j)}{\partial x}=\frac{1}{2}\left(\frac{\sigma_{xx}(i+1/2,j)-\sigma_{xx}(i,j)}{\frac{1}{2}\text{Δ}x}+\frac{\sigma_{xx}(i,j)-\sigma_{xx}(i-1/2,j)}{\frac{1}{2}\text{Δ}x}\right)\\ \;=\frac{\sigma_{xx}(i+1/2,j)-\sigma_{xx}(i,j)}{\text{Δ}x}+\frac{\sigma_{xx}(i,j)-0}{\text{Δ}x}\\ \;=\frac{\sigma_{xx}(i+1/2,j)}{\text{Δ}x}\\ \;=\frac{c_{1}\frac{\partial u_{x}}{\partial x}(i+1/2,j)+c_{2}\frac{\partial u_{y}}{\partial y}(i+1/2,j)}{\text{Δ}x}\\ \;=\frac{c_{1}(i+1,j)+c_{1}(i,j)}{2}\frac{\left[u_{x}(i+1)\;-u_{x}(i)\right]}{\text{Δ}x^{2}}\\ \;+{\tilde{c}}_{2}\frac{u_{y}(i,j+1)+u_{y}(i+1,j+1)-u_{y}(i,j-1)-u_{y}(i+1,j-1)}{4\text{Δ}x\text{Δ}y}, \end{array}\;(25)$$

with

$${\tilde{c}}_{2}=\frac{c_{2}(i,j+1)+c_{2}(i+1,j+1)+c_{2}(i,j-1)+c_{2}(i+1,j-1)+2c_{2}(i,j)+2c_{2}(i+1,j)}{8},\;(26)$$

whereas the second term becomes

$$\;\frac{\partial \sigma_{xy}(0,j)}{\partial y}=\frac{\sigma_{xy}(0,j+1)-\sigma_{xy}(0,j-1)}{2\text{Δ}y}\approx 0.\;(27)$$

Note that the boundary condition formally states that *σ _xy_* ( *−*1 */*2 *, j*) = 0, whereas this is approximated by *σ _xy_* (0 *, j*) = 0 in the numerical implementation. A possible solution to circumvent this approximation is to use a staggered grid where one of the grids contains the displacement values and the other one the stress values. Another solution could be to implement an additional raster of mesh points on the outside of the “original” mesh that can be used to set the proper boundary conditions. In this extension, we have chosen to use the approximation *σ _xy_* (0 *, j*) *≈ σ _xy_* ( *−*1 */*2 *, j*) = 0 because the mesh is already defined and fixed for the magnetization. However, using another or an additional mesh would strongly complicate the problem since the magnetoelastic extension is built based on the M uM ax3 framework, which is designed to work with one mesh only. 

The *y*-component of the elastic body force is given by

$$\;f_{\text{el,}y}=\frac{\partial \sigma_{yx}(0,j)}{\partial x}+\frac{\partial \sigma_{yy}(0,j)}{\partial y}\;.\;(28)$$

Considering again that the surface of the magnetic material is located at *i* = *−*1 */*2 and taking into account the boundary conditions given in Equation (23), the first term can be rewritten as

$$\begin{array}{l} \frac{\partial \sigma_{xy}(i=0,j)}{\partial x}=\frac{\sigma_{xy}(i+1/2,\;j)-\sigma_{xy}(i,\;j)}{\text{Δ}x}\;+\frac{\sigma_{xy}(i,\;j)-\sigma_{xy}(i-1/2,\;j)}{\text{Δ}x}\\ \;=\frac{\sigma_{xy}(i+1/2,\;j)-\sigma_{xy}(i,\;j)}{\text{Δ}x}\;+\frac{\sigma_{xy}(i,\;j)-0}{\text{Δ}x}\\ \;=\frac{\sigma_{xy}(i+1/2,\;j)}{\text{Δ}x}\\ \;=\frac{c_{4}(i,j)}{\text{Δ}x}\left(\frac{\partial u_{x}}{\partial y}(i+1/2,\;j)+\frac{\partial u_{y}}{\partial x}(i+1/2,\;j)\right)\\ \;=\frac{{\tilde{c}}_{4}}{2\text{Δ}x\text{Δ}y}\left(\frac{u_{x}(i,\;j+1)+u_{x}(i+1,\;j+1)}{2}-\frac{u_{x}(i,\;j-1)+u_{x}(i+1,\;j-1)}{2}\right)\\ \;+\;\frac{c_{4}(i,j)+c_{4}(i+1,j)}{2}\frac{1}{\text{Δ}x^{2}}(u_{y}(i+1,\;j)-u_{y}(i,\;j))\\ \;=\frac{{\tilde{c}}_{4}}{4\text{Δ}x\text{Δ}y}\left(u_{x}(i,\;j+1)+u_{x}(i+1,\;j+1)-u_{x}(i,\;j-1)-u_{x}(i+1,\;j-1)\right)\\ \;+\;\frac{c_{4}(i,j)+c_{4}(i+1,j)}{2\text{Δ}x^{2}}(u_{y}(i+1,\;j)-u_{y}(i,\;j)) \end{array}\;(29)$$

and the second term as

$$\begin{array}{l} \frac{\partial \sigma_{yy}(i=0,j)}{\partial y}=c_{1}\;(i,j)\frac{\partial^{2}u_{y}}{\partial y^{2}}(i,j)+c_{2}\;(i,j)\frac{\partial^{2}u_{x}}{\partial x\partial y}(i,j)\\ \;=\frac{c_{1}\;(i,j+1)+2c_{1}(i,j)+c_{1}\;(i,j-1)}{4\text{Δ}y^{2}}(u_{y}(i,j+1)+u_{y}(i,j-1)-2u_{y}(i,j))\\ \;+\frac{{\tilde{c}}_{2}}{2\text{Δ}y}\left(\frac{\partial u_{x}}{\partial x}(i,j+1)-\frac{\partial u_{x}}{\partial x}(i,j-1)\right)\\ \;=\frac{c_{1}\;(i,j+1)+2c_{1}(i,j)+c_{1}\;(i,j-1)}{4\text{Δ}y^{2}}(u_{y}(i,j+1)+u_{y}(i,j-1)-2u_{y}(i,j))\\ \;+\frac{{\tilde{c}}_{2}}{2\text{Δ}y\text{Δ}x}\left(u_{x}(i+1,\;j+1)-u_{x}(i,j+1)-[u_{x}(i+1,\;j-1)-u_{x}(i,\;j-1)]\right)\\ \;=\frac{c_{1}\;(i,j+1)+2c_{1}(i,j)+c_{1}\;(i,j-1)}{4\text{Δ}y^{2}}(u_{y}(i,j+1)+u_{y}(i,j-1)-2u_{y}(i,j))\\ \;+\frac{{\tilde{c}}_{2}}{2\text{Δ}y\text{Δ}x}(u_{x}(i+1,\;j+1)-u_{x}(i,j+1)-u_{x}(i+1,\;j-1)-u_{x}(i,\;j-1)). \end{array}\;(30)$$

Finally, the *z*-component of the elastic body force is given by

$$\;f_{\text{el,}z}=\frac{\partial \sigma_{zx}(0,j)}{\partial x}+\frac{\partial \sigma_{zy}(0,j)}{\partial y}\;.\;(31)$$

In this case, the first term can be rewritten as

$$\;\begin{array}{l} \frac{\partial \sigma_{zx}(0,j)}{\partial x}=\frac{\sigma_{zx}(i+1/2,\;j)-\sigma_{zx}(i-1/2,\;j)}{\text{Δ}x}\\ \;=\frac{\sigma_{zx}(i+1/2,\;j)}{\text{Δ}x}\\ \;=\frac{c_{4}(i+1/2,\;j)}{\text{Δ}x}\frac{\partial u_{z}}{\partial x}(i+1/2,\;j)\\ \;=\frac{c_{4}(i+1,\;j)+c_{4}(i,\;j)}{2}\frac{u_{z}(i+1,\;j)-u_{z}(i,\;j)}{\text{Δ}x^{2}} \end{array}\;(32)$$

and the second term as

$$\;\begin{array}{l} \frac{\partial \sigma_{zy}(0,j)}{\partial y}=c_{4}(i,\;j)\frac{\partial^{2}u_{z}}{\partial y^{2}}(i,\;j)\\ \;={\tilde{c}}_{4}\frac{u_{z}(i,\;j+1)-2u_{z}(i,\;j)+u_{z}(i,\;j-1)}{\text{Δ}y^{2}}. \end{array}\;(33)$$

Within M uM ax3, all these mathematical operations are parallelized and performed on a GPU to reduce the computational time.

### Operation of the magnetoelastic extension

The instructions to install the software are provided on the M uM ax3 github page https://github.com/mumax/ The software requires the installation of Go and the CUDA Toolkit as well as the availability of an NVIDIA GPU.

In M uM ax3, several different data types exist, such as parameters, fields, excitations, *etc.*, which can be defined, modified, or extracted via various methods ^52, 68^. This section presents an overview of additional material parameters, vector fields, excitations, energies, boundary conditions, and initial states that are defined in the magnetoelastic extension. Their usage is analogous to the usage of the equivalent elements defined in M uM ax3 ^52, 68^ and will therefore be only briefly discussed. Note that all regular M uM ax3 methods can also be applied to the new elements defined in the M uM ax3 extension.

***New material parameters*.** The usage and the assignment of the newly defined material parameters are the same as for traditional M uM ax3 material parameters. Details can be found in the M uM ax3 documentation ^68^. Parameters can depend on time and—through the definition of regions—on position. The following list provides an overview of novel material parameters and their units, which are defined in the magnetoelastic extension of M uM ax3:

- C11: stiffness constant *c*_11_ = *c*_22_ = *c*_33_ of the stiffness tensor with unit [Nm ^*−*2^];
- C12: stiffness constant *c*_12_ = *c*_13_ = *c*_23_ of the stiffness tensor with unit [Nm ^*−*2^];
- C44: stiffness constant *c*_44_ = *c*_55_ = *c*_66_ of the stiffness tensor with unit [Nm ^*−*2^];
- eta: Phenomenological elastic damping constant with unit [kgs ^*−*1^m ^*−*3^];
- rho: Mass density with unit [kgm ^*−*3^];
- B1: first magnetoelastic coupling constant with unit [Jm ^*−*3^];
- B2: second magnetoelastic coupling constant with unit [Jm ^*−*3^].

***New vector fields*.** There are two vector field types in M uM ax3. The first one is called a *variable* and can be treated in the same way as the magnetization. The second type is called a *quantity* and represents a field that can be derived from the fundamental *variables*. The usage of the new vector fields is the same as for the traditional M uM ax3 ^68^. The novel *variables* and their units defined within the magnetoelastic extension of M uM ax3 are:

- u: elastic displacement vector with unit [m];
- du: velocity vector with unit [ms ^*−*1^];

The novel *quantities* and their units defined within the magnetoelastic extension of M uM ax3 are:

- normStrain: vector that contains the normal strain components [ *ε _xx_, ε _yy_, ε _zz_*], calculated according to $\hat{\varepsilon }=\frac{1}{2}\left(\nabla u+{\left(\nabla u\right)}^{T}\right)$ , with unit [Nm ^*−*2^]. Note that the strain corresponds to the real strain and not the engineering strain;
- shearStrain: vector that contains the shear strain components [ *ε _xy_, ε _yz_, ε _xz_*], calculated according to $\hat{\varepsilon }=\frac{1}{2}\left(\nabla u+{\left(\nabla u\right)}^{T}\right)$ , with unit [Nm ^*−*2^]. Again, note that the strain corresponds to the real strain and not the engineering strain;
- normStress: vector that contains the normal stress components [ *σ _xx_, σ _yy_, σ _zz_*], calculated according to Hooke’s law, $\hat{\sigma }=\hat{c}\hat{\varepsilon }$ , with unit [Nm ^*−*2^];
- shearStress: vector that contains the shear stress components [ *σ _xy_, σ _yz_, σ _xz_*], calculated according to Hooke’s law, with unit [Nm ^*−*2^];
- poynting: elastic Poynting vector, calculated via *P*_el_ = $-\hat{\sigma }v$ , with unit [Wm ^*−*2^];
- B_mel: magnetoelastic field *µ*_0_ ***H***_mel_ with unit [T] implemented according to Equation (3);
- F_mel: magnetoelastic body force with unit [Nm ^*−*3^] implemented according to Equation (3).

The magnetoelastic M uM ax3 extension can simulate magnetoelastodynamics for materials with cubic or higher symmetries. Hence, the module only contains the *c*_11_, *c*_12_, and *c*_44_ stiffness constants. For the strain, the magnetoelastic field, and the magnetoelastic body force, standard central finite difference schemes are used to calculate the derivatives in the respective equations.

***Excitations*.** Excitations, such as applied fields and current densities, can depend on time and space following the form *f*( *t*) *× g*( *x, y, z*). Here, *g* can be a continuously varying spatial profile. This is different from the material parameters, which need to be uniform within each region. The following gives an overview of novel excitations and their units within the magnetoelastic M uM ax3 extension. Their usage is analogous to the regular M uM ax3 usage for excitations ^68^:

- force_density: external body force ***f***_ext_ that is added to the effective body force ***f***_eff_ = ***f***_el_ + ***f***_mel_ + ***f***_ext_, with unit [Nm ^*−*3^];
- exx: *ε _xx_* strain component;
- eyy: *ε _yy_* strain component;
- ezz: *ε _zz_* strain component;
- exz: *ε _xz_* strain component;
- exy: *ε _xy_* strain component;
- eyz: *ε _yz_* strain component.

***Energies*.** Energies can be extracted at different moments in time to study the system. Every energy contribution can be extracted as either a field or a scalar value. The field corresponds to the energy density and the scalar object corresponds to the total energy of that contribution inside the system. The following list gives an overview of novel energies and their units defined in the magnetoelastic M uM ax3 extension:

- Edens_el: elastic energy density (unit [Jm ^*−*3^]) in the linear regime where Hooke’s law is valid, implemented via

$$\;\varepsilon_{\text{el}}=\frac{1}{2}\hat{\sigma }:\;\hat{\varepsilon }=\frac{1}{2}\sum_{i,j,k,l}c_{ijkl}\varepsilon_{kl}\varepsilon_{ij};\;(34)$$

- E_el: the total elastic energy, *i.e.* $\int \varepsilon_{\text{el}}dV$ , with unit [J];

- Edens_mel: magnetoelastic energy density (unit [Jm ^*−*3^]) for a material with cubic (or higher) crystal symmetry, implemented via

$$\;\varepsilon_{mel}=B_{1}\sum_{i}m_{i}^{2}\varepsilon_{ii}+B_{2}\sum_{i\neq j}m_{i}m_{j}\varepsilon_{ij};\;(35)$$

- E_mel: total magnetoelastic energy, *i.e.* $\int \varepsilon_{\text{mel}}dV$ , with unit [J];

- Edens_kin: kinetic energy density (unit [Jm ^*−*3^]), implemented via

$$\;\varepsilon_{\text{kin}}=\frac{\rho {\left\|v\right\|}^{2}}{2};\;(36)$$

- E_kin: total kinetic energy, *i.e.* $\int \varepsilon_{\text{kin}}dV$ , with unit [J].

***Initial states*.** Three new initial states have been implemented for both magnetization and displacement. Their usage is equivalent to the standard M uM ax3 usage for the definition of the initial state ^68^.

- A Gaussian pulse with spherical distribution of the out-of-plane vector component: GaussianSpherical_outplane(A, pos_x, pos_y, sig_x, sig_y float64), implemented as

$$\;\begin{array}{l} u_{x}=0\\ u_{y}=0\\ u_{z}=Ae^{-\frac{{\left(x-x_{0}\right)}^{2}}{2\sigma_{x}^{2}}\frac{{\left(y-y_{0}\right)}^{2}}{2\sigma_{y}^{2}}}, \end{array}\;(37)$$

with *x*_0_ = pos_x, *y*_0_ = pos_y, *σ _x_* = sig_x and *σ _y_* = sig_y;

- A Gaussian pulse with spherical (symmetric) distributions of the in-plane vector components: GaussianSpherical(A, pos_x, pos_y, sig_x, sig_y, angle float64), implemented as

$$\;\begin{array}{l} u_{x}=\cos(\theta )Ae^{-\frac{{\left(x-x_{0}\right)}^{2}}{2\sigma_{x}^{2}}\frac{{\left(y-y_{0}\right)}^{2}}{2\sigma_{y}^{2}}}\\ u_{y}=\sin(\theta )Ae^{-\frac{{\left(x-x_{0}\right)}^{2}}{2\sigma_{x}^{2}}\frac{{\left(y-y_{0}\right)}^{2}}{2\sigma_{y}^{2}}}\\ u_{z}=0, \end{array}\;(38)$$

with *θ* = angle in degrees, *x*_0_ = pos_x, *y*_0_ = pos_y, *σ _x_* = sig_x and *σ _y_* = sig_y;

- Uniformly oriented in-plane vector components along a specific direction with a Gaussian distribution along the transverse direction GaussianUniform(A, pos, sig, angle1, angle2 float64), implemented as

$$\;\begin{array}{l} u_{x}=\cos(\theta )Ae^{-\frac{{\left(x^{\prime }-x_{0}\right)}^{2}}{2\sigma^{2}}}\\ u_{y}=\sin(\theta )Ae^{-\frac{{\left(x^{\prime }-x_{0}\right)}^{2}}{2\sigma^{2}}}\\ u_{z}=0, \end{array}\;(39)$$

with *θ* = angle2 in degrees, *x ^'^* = cos( *ϕ*) *x* + sin( *ϕ*) *y*, *ϕ* = angle1, *x*_0_ = pos and *σ* = sig.

***Boundary conditions*.** It is possible to apply several types of elastic boundary conditions in the new magnetoelastic M uM ax3 module. All elastic boundary conditions correspond to interfaces in the *x*- and *y*-directions of the film since uniform displacement is assumed along the *z*-direction.

- Free boundary conditions, corresponding to zero forces at the edges of the film. These are the default boundaries in the *x*- and *y*-directions of the film.
- Periodic boundary conditions, which can be enabled in the same way as in the traditional M uM ax3 framework via the SetPBC() command ^68^.
- Absorbing boundaries, which can be implemented by defining regions with gradually increasing elastic damping *η*, similarly to the gradually increasing damping used to absorb spin waves in a purely magnetic system ^70^. This is, for example, useful to prevent wave reflection at surfaces or interfaces.

Fixed boundary conditions for the displacement are also possible. Such boundary conditions can be obtained by defining regions at the edges of the mesh with fixed displacement values. The parameter frozenDispLoc defines a region with fixed displacement, and the parameter FrozenDispVal defines the fixed displacement value. This works in the same way as the regular frozenspins function in M uM ax3, which fixes the spins in a specific region. However, spin (magnetic moment) has always the same norm, whereas the norm can vary for the displacement. Therefore, the additional function FrozenDispVal has been defined. Note that this value can be time dependent and thus can also act as an excitation source.

For example, the code


defregion(2,rect(400e-9,
     1000e-9).transl(-1500e-9,-1000e-9,0))
frozenDispLoc.SetRegion(2,1)
frozenDispVal.SetRegion(2,0,0,0))


sets region 3 to zero displacement. It is also possible to define a time-varying displacement in this region:


defregion(2,rect(400e-9,
     1000e-9).transl(-1500e-9,-1000e-9,0))
frozenDispLoc.SetRegion(2,1)
frozenDispVal.SetRegion(2,
     vector(0.1e-13*sin(2*3.1415*1e9*t),0,0))


This code therefore acts as as a source of (magnetoelastic) waves. 

## Use cases

The magnetoelastic module has been extensively quantitatively tested. A demonstration of its capabilities has been published in Ref. 71, which discusses simulations of confined magnetoelastic waves in nanoscale magnetostrictive CoFeB waveguides. Below, we reproduce verbatim the input script (including annotations) used to generate the data in Figure 7 in Ref. 71 as an example. As output, the input script generates data in the .ovf format. The . ovf format is conventionally used by micromagnetic simulators, including M uM ax3 ^52^ and OOMMF ^49^, and documented in detail in the respective manuals.


//Specify output format
OutputFormat = OVF2_TEXT



//mesh
dx := 5e-9
dy := 5e-9
dz := 20e-9
Nx := 4000
Ny := 40
Nz := 1
SetMesh(Nx, Ny, Nz, dx, dy, dz, 0, 0, 0)



//External parameters
f      := 9.8e9
Bac := 1e-3
Bdc := 5e-3



//Elastic parameters CoFeB
C11 = 283e9
c12 = 166e9
C44 = 58e9
rho = 8e3
eta = 0



//Magnetoelastic parameters CoFeB
B1 = -8.8e6
B2 = -8.8e6



//Magnetic parameters CoFeB
Msat = 1.2e6
Aex  = 18e-12
alpha = 4e-3



//Absorbing regions on both sides
//Left
defregion(24,xrange(-5.2e-6,-5e-6))
eta.setregion(24,1e12)
alpha.setregion(24,1e-2)
defregion(25,xrange(-5.4e-6,-5.2e-6))
eta.setregion(25,3e12)
alpha.setregion(25,5e-2)
defregion(26,xrange(-5.6e-6,-5.4e-6))
eta.setregion(26,6e12)
alpha.setregion(26,1e-1)
defregion(27,xrange(-Inf,-5.6e-6))
eta.setregion(27,5e13)
alpha.setregion(27,0.5)
//right
defregion(14,xrange(5e-6,5.2e-6))
eta.setregion(14,1e12)
alpha.setregion(14,5e-2)
defregion(15,xrange(5.2e-6,5.4e-6))
eta.setregion(15,3e12)
alpha.setregion(15,5e-2)
defregion(16,xrange(5.4e-6,5.6e-6))
eta.setregion(16,6e12)
alpha.setregion(16,1e-1)
defregion(17,xrange(5.6e-6,Inf))
eta.setregion(17,5e13)
alpha.setregion(17,0.5)



//Static field
B_ext = vector(Hdc, 0, 0)



//Excitation field
defregion(2,xrange(-50e-9,50e-9))
B_ext.setregion(2,
   vector(Hdc, Hac*sin(2*pi*f*t), 0))



//Initial state
u=uniform(0,0,0)
m=uniform(1,0,0)



//Solver
SetSolver(9)
fixdt = 1e-13



//Output
autosave(B_mel, 1e-9)
autosave(F_mel, 1e-9)
autosave(m, 1e-9)
autosave(u, 1e-9)
autosave(normstrain, 1e-9)
autosave(shearstrain, 1e-9)
autosave(B_demag, 1e-9)



//running
run(10.0e-9)


To obtain an estimate of the additional required computation time due to the magnetoelastic extension, we executed this script on an NVIDIA Quadro K2200 GPU for cases where magnetoelastic interaction is present, *i.e.* using Setsolver(9), and without magnetoelastic interaction, *i.e.* using Setsolver(5). Including the magnetoelastic interactions, the simulation took 54:31 min. By contrast, the simulation time was 38:14 min when the magnetoelastic coupling was deactivated.

## Conclusion

This paper describes an extension of the established finite difference micromagnetic solver M uM ax3, which adds capabilities to calculate elastodynamics including the magnetoelastic coupling between mechanical and magnetic degrees of freedom. It therefore allows for the finite difference simulation of magnetoelastodynamics in 1D, 2D or quasi-3D systems. The implementation in MuMax3 means that all standard M uM ax3 functionalities can also be used in the magnetoelastic extension, and that the mathematical operations can be performed on GPUs, resulting in low computational times. Multiple different boundary conditions have been implemented, specifically periodic, fixed, and free bounadry conditions, as described in Sec. . The software is freely available under the GNU General Public License v3.

## Data availability

All data underlying the results are available as part of the article and no additional source data are required.

## Software availability

The M uM ax3 magnetoelastic extension is open source and freely available in the github repository. The source files can be found in the mumax/engine and mumax/cuda subfolders and have the term elastic in their file name.

- Source code available from: https://github.com/Fredericvdv/Magnetoelasticity_MuMax3
- Archived source code at time of publication: https://doi.org/10.5281/zenodo.4450141
- License: GNU General Public License v3
