# Supplementary material for: Finite difference magnetoelastic simulator
Source: Open Res Eur. 2021 Apr 19;1:35. [Version 1] doi: 10.12688/openreseurope.13302.1 (PMC10445870; doi:10.12688/openreseurope.13302.1)
Supplement: Peer review history

# Peer review: Reviewer response for version 1

**DOI:** 10.21956/openreseurope.14372.r26736

**Published:** 2021-05-12

**Authors:** Martí Gich

**Affiliations:** 1 Institut de Ciència de Materials de Barcelona ICMAB, Consejo Superior de Investigaciones Científicas CSIC, Bellaterra, Spain

This work reports on a magnetoelastic extension to enhance the capabilities of the free MuMax3 micromagnetics simulation code. It fills an unmet need, providing open access simulation capabilities to support the increasing number of studies in which the magnetostrictive response is relevant. Indeed, the paper introduction provides a good overview of the state of the art and the need of these simulations in the context of magnetoelectric spintronic devices.

 The fundamental equations in which the code is based, its implementation in MuMax3 and considerations on the treatment of elastic boundary conditions are discussed with clarity and simplicity, making the paper understandable to non-specialists in simulations and mechanics of solids, among which I include myself. It is particularly welcome the section entitled *Operation of the magnetoelastic extension*, giving useful practical information and details for making it easier for new users to getting started in the new software, which is not always the case in the literature describing new simulation codes. In a similar vein, the authors provide the commented input script of the magnetoelastic module which was used to simulate a nanoscale magnetostrictive CoFeB waveguide for one of their recent publications.

 Overall, this new module is poised to become a valuable support for research on a range of systems and applications in which magnetostriction can be relevant and thus the manuscript should be indexed on Open Research Europe.

 However, I think that a short discussion on the limitations of the module would improve the manuscript. More specifically, **i)** The code has been implemented for cubic crystal symmetries. Since many relevant magnetic materials have lower symmetries than the cubic one it would be interesting a comment on the possible extensions to lower symmetries and in particular to systems with uniaxial anisotropy. **ii)** Since the full 3D elastodynamics description assumes of uniform displacement along the z direction, it would be helpful some discussion on the limitations of this assumption and on how to assess, using the module, how realistic this is for a particular model, if this is possible.

 Other additional minor remarks:

 Page 4, end of line 16: “solve” should be replaced by “solving”.

 At the end of page 7, mention is made to eq. 6. Since it is indeed eq. 7, a simplification of eq. 6 considering uniform displacement along z, which is used in the code, to avoid confusion in the discussion of boundary conditions that follows it would be better to refer to eq. 7.

 At the end of page 14 the text description accompanying the example of defining a time dependent displacement boundaries refers to “region 3” but it seems that the code is rather for a region 2…

 The paper repeatedly refers to “cubic (or higher) crystal symmetry”. To my understanding this is a bit confusing because the cubic one is the highest of all the crystal symmetries. A material which has a higher symmetry than that of a cubic crystal must be polycrystalline and therefore it is not strictly a crystal but a polycrystalline material.

Is the rationale for developing the new software tool clearly explained?

Yes

Is the description of the software tool technically sound?

Yes

Are sufficient details of the code, methods and analysis (if applicable) provided to allow replication of the software development and its use by others?

Yes

Is sufficient information provided to allow interpretation of the expected output datasets and any results generated using the tool?

Yes

Reviewer Expertise:

Synthesis of oxides, magnetic and structural characterization of oxides.

I confirm that I have read this submission and believe that I have an appropriate level of expertise to confirm that it is of an acceptable scientific standard.

# Peer review: Reviewer response for version 1

**DOI:** 10.21956/openreseurope.14372.r26735

**Published:** 2021-05-12

**Authors:** Mathias Weiler

**Affiliations:** 1 TU Kaiserslautern, Kaiserslautern, Germany

The authors developed an open-source extension to the finite difference simulation software MuMax3 that allows simulating dynamic magneto-elastic interactions. MuMax3 is widely used for simulation of magnetization dynamics due to its open-source availability, ease of use and fast computation times. Magneto-elastic interactions are generally relevant for magnetization dynamics as most magnetic materials are magneto-elastic. Magneto-elasticity can enable magnon-phonon hybridization. This software extension should be a very powerful tool to simulate magneto-elastic interactions and hybrid magnon-phonon dynamics beyond the limits of simple analytical models. Previous open-source software implementations have only been able to simulate the effect of magneto-elastics on magnetization dynamics, but not the back-action of magnetization dynamics onto the elastics. Considering this back-action is however required for accurate description of magneto-elastic phenomena. The contribution is thus of high relevance and novelty for the field.

 The description of the underlying physics and parameters used in the code is clear and the authors introduce the relevant background to motivate their work in a suitable manner. I found this to be a very high quality and accessible work that will be important in particular for scientists intending to employ and tailor the extension for their own problems.

 As a minor suggestion for clarification, the authors should consider providing a short description of the limitations of the extension. The authors demonstrated in Ref. 71 that the extension can be used to simulate confined magneto-elastic waves in thin waveguides. It would be nice to briefly mention here if there are other geometries where their extension can (or cannot) be used. As an example, many researchers are interested in the interaction of surface acoustic waves (SAWs) and magnetization dynamics in magnetic thin films. The typical system thereby is a substrate / thin magnetic film bilayer. To me it is not clear to what capacity the extension can be used as is or modified to address magneto-elastic phenomena in this configuration. For SAW-spin wave interactions in laterally extended systems, is it possible to work around the restriction of uniform displacement in the z direction by taking a coordinate system with SAW propagation along x and interface normal along y (plane surface waves)? A major challenge might be accurate simulation of SAWs that dominantly propagate in the non-magnetic substrate but still interact with spin waves in the magnetic film. Can the authors comment on this challenge?

 As a second point for potential future modifications, it would be interesting to discuss further mechanisms that can lead to a coupling of magnetization dynamics and strain even in the absence of magneto-elasticity. In this regard, Xu and coworkers ^1^ recently discussed magneto-rotation coupling which would add an additional term to Eq. (3) stemming from the rotation of the magnetic anisotropy axis due to shear strain. This coupling can be comparable or even larger than magneto-elastic coupling ^1^^,^^2^. Is it in principle possible to extend the code to consider magneto-rotation coupling?

Is the rationale for developing the new software tool clearly explained?

Yes

Is the description of the software tool technically sound?

Yes

Are sufficient details of the code, methods and analysis (if applicable) provided to allow replication of the software development and its use by others?

Yes

Is sufficient information provided to allow interpretation of the expected output datasets and any results generated using the tool?

Yes

Reviewer Expertise:

Experimental solid state physics, magnetism, magnetization dynamics.

I confirm that I have read this submission and believe that I have an appropriate level of expertise to confirm that it is of an acceptable scientific standard.

## References

[1] : Nonreciprocal surface acoustic wave propagation via magneto-rotation coupling. *Sci Adv* .2020;6(32) : 10.1126/sciadv.abb1724 eabb1724 10.1126/sciadv.abb1724 32821833PMC7413730

[2] : Nonreciprocal Dzyaloshinskii-Moriya Magnetoacoustic Waves. *Phys Rev Lett* .2020;125(21) : 10.1103/PhysRevLett.125.217203 217203 10.1103/PhysRevLett.125.217203 33275006

# Peer review: Reviewer response for version 1

**DOI:** 10.21956/openreseurope.14372.r26749

**Published:** 2021-05-05

**Authors:** Greg Carman

**Affiliations:** 1 Department of Mechanical and Aerospace Engineering, University of California, Los Angeles, Los Angeles, CA, USA

I believe that the authors have done a wonderful job of expanding the availability of software to address the important applications of magnetoelastic problems. To my knowledge the only other approaches are housed in Finite Element based programs such as Comsol which requires the partial differential equations to be input AND the structure is fairly slow and limited in size of problems that can be solved. Below, I provide some comments the authors may wish to consider adding or commenting on in the manuscript.

1. Can authors comment on how this code which I believe is FDTD based compares with FEM based approaches. I am hopeful that the authors know the answer and can provide comparison metrics so that others working in this area can make informed decisions on which numerical platform for this problem is superior.
2. Authors reduce the full 3D problem to a subset with the assumption of zero strain in the z direction, eq 7. I am not sure why they do this or is it because the full 3D problem is to cumbersome to solve. The previous FEM solutions in the literature do not make this assumption. Adding a sentence or two describing why this assumption is made will help the community.

Is the rationale for developing the new software tool clearly explained?

Yes

Is the description of the software tool technically sound?

Yes

Are sufficient details of the code, methods and analysis (if applicable) provided to allow replication of the software development and its use by others?

Yes

Is sufficient information provided to allow interpretation of the expected output datasets and any results generated using the tool?

Yes

Reviewer Expertise:

Magnetoelectric and multierroic.

I confirm that I have read this submission and believe that I have an appropriate level of expertise to confirm that it is of an acceptable scientific standard.
